# Risk Factors of Orofacial Pain: A Population-Based Study in West Java Province, Indonesia

**DOI:** 10.2174/1874210601711010710

**Published:** 2017-12-29

**Authors:** Rasmi Rikmasari, Gilang Yubiliana, Tantry Maulina

**Affiliations:** 1 Prosthodontic Department, Faculty of Dentistry, Universitas Padjadjaran, Bandung, Indonesia; 2 Community Dental Health Department, Faculty of Dentistry, Universitas Padjadjaran, Bandung, Indonesia; 3 Oral Surgery Department, Faculty of Dentistry, Universitas Padjadjaran, Bandung, Indonesia

**Keywords:** Orofacial pain, Risk factors, Oral parafunctional habits, Indonesia, Temporomandibular Disorders (TMD), Unilateral chewing

## Abstract

**Background:**

The management of orofacial pain in Indonesia has not been well performed, which consequently led to an increase in the orofacial pain occurrences and a decreased quality of life. One of the possible reasons for this particular matter is the lack of evaluation on the risk factors that might induce orofacial pain in some individuals.

**Objective:**

The objective of the current study was to evaluate the risk factors of orofacial pain on productive age population in West Java province, Indonesia.

**Methods:**

One thousand and fifty-six participants (522 males; 534 females) were recruited for the study. A questionnaire that consists of demographic questions and questions evaluating several assumed risk factors for orofacial pain was used in a single interview. All data was analyzed by using Chi Square test to test the significance, Odds Ratio (OR), as well as Relative Risk (RR) by using *SPSS* version 23 (IBM Statistic, USA).

**Results:**

The result of the current study revealed that bruxism (*p*<0.01), daytime clenching (*p*<0.01), and unilateral chewing (*p*<0.01) were significantly related to the occurrence of orofacial pain. It was also found that participants who performed multitude of heavy liftings at work have an increased risk (RR=1.19: 95% CI: 1.04 – 1.35) of having orofacial pain compared to those who do not.

**Conclusion:**

Risk factors for the occurrence of orofacial pain on productive age population in Indonesian sample consisted of oral parafunctional habits and non-parafunctional habits, such as heavy lifting. Further study in this particular topic is of importance.

## 1 INTRODUCTION

Orofacial pain is defined as pain occurring in the soft tissue as well as hard tissue of the head, including the oral region, face, and neck [1]. The complex structure of the oral and facial region has madeorofacial pain as oneof the challenging conditions to be treated by clinicians [2]. The fact that it impacted the patient’s quality of life and caused high level of impairment [3-6] has been one of the highlights in the management of orofacial pain. Amongst the groups of patients who were affected by orofacial pain, the age group of 18-45 years old, is a productive age group that is mostly affected by orofacial pain in regards to work productivity. A study about orofacial pain and its associated impact on the community showed that this particular age group showed a high prevalence of orofacial pain [7]. Whilst previous studies about the impact of orofacial pain on work activities showed significant correlations between the duration and intensity of orofacial pain and impaired work activities [8, 9].

There are several factors that are considered to be the risk factors for orofacial pain. A model developed in a study by Maixner *et al*, (2011). based on a model proposed by Dworkin *et al*, (1992) about the biopsychosocial model of Temporomandibular Disorders (TMD) revealed that TMD and its pain and other accompanying symptoms were influenced proximally by two factors, psychological distress and pain amplification, with the later factor includes pro-inflammatory states, impaired pain regulation, cardiovascular function, and neuroendocrine function [10, 11]. Aside from these two assumed risk factors of orofacial pain, the two factors also assumed to be associated with the occurrence of orofacial pain are age and gender. In a study about gender difference in the occurrence of TMD, Bagis *et al*, (2012) showed that pain in the masseter muscle as well as TMJ pain while resting was found to be more frequent in female than male participants. In this study, age was also found to have a significant effect on the occurrence of TMD [12].

Another study by Anggarwai *et al*, (2010) about the risk factors for the onset of chronic orofacial pain on adults aged 18-75 years that were patients from a general practice in the North West of England revealed that grinding, anxiety, depression, health anxiety, chronic widespread pain and irritable bowel syndrome were the factors that were significantly (*p* < 0.05) associated to the incidence of chronic orofacial pain. In this population-based prospective study that involved 1329 participants, it was also revealed that age was a risk factor for the onset of chronic orofacial pain, with those who are in the low age category is being the group that is more prone to chronic orofacial pain [13].

The variation of results in previous studies about the risk factors of orofacial pain as well as the high prevalence of orofacial pain in Indonesian sample in previous studies [8,14] emphasizes the need of a conduction of an epidemiological study that can evaluate the risk factors for the occurrence of orofacial pain in Indonesia. Therefore, the aim of the current study was to identify the risk factors of orofacial pain in the productive age group in Indonesia and associate these risk factors to the occurrence of orofacial pain.

## 2 MATERIALS AND METHODS

The current study randomly recruited 1056 (522 males; 534 females) participants from six regencies and three cities from West Java province that were selected by using the Cluster Sampling method. After the cities and regencies were selected, participants who were at the age of 18 - 45 years old were randomly selected by using the Simple Random Sampling (SRS) method. Prior to the start of the study, an ethical approval was gained from the Health Research Ethic Committee Faculty of Medicine Universitas Padjadjaran, Bandung, Indonesia. All procedures in the current study has been conducted in full accordance to the Declaration of Helsinski and prior to the start of data collection, all participants signed a written informed consent. All participants’ demographical data that includes educational attainment (elementary school, junior high school, senior high school, college (3 years of higher education that resulted in a Diploma degree)and university (5 years of education that resulted in a Bachelor degree)), type of occupation (1: private sector worker; 2: entrepreneur; 3: laborer; 4: driver; 5: housewife; 6: government employee; 7: student; 8: unemployed; 9: health professional), as well as age and gender were recorded in Table **1**. Two calibrated interviewers interviewed the participants by using a validated questionnaire in Bahasa Indonesia, which is the official language of Indonesia.

**Fig. (1):**
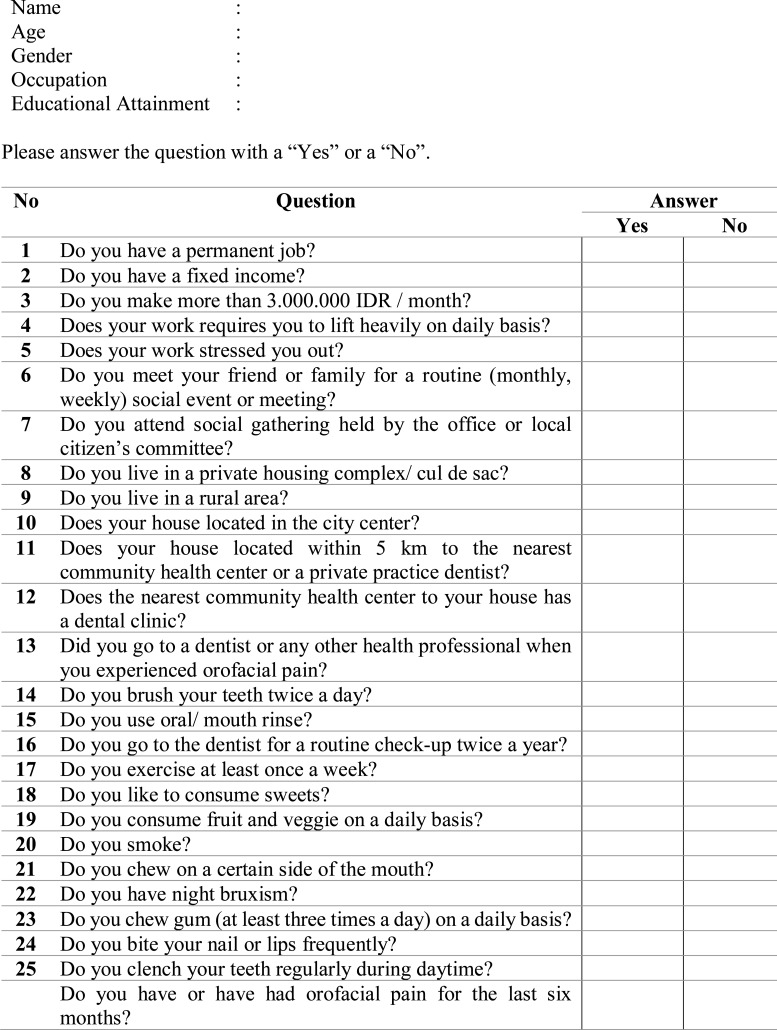
Orofacial pain risk factors questionnaire (English version).

The questionnaire consists of twenty-five questions (Fig. **1**) that evaluated several factors which were assumed to be the risk factors for orofacial pain, and one main question that evaluated the occurrence of orofacial pain within the last six months.Orofacial pain included in the current study is the pain occurring in the tooth (toothache), pain in the jaw joint/s, pain in the area just in front of the ear/s, pain in or around the eyes, pain when opening the mouth wide, shooting pains in the face or cheeks, pain in the jaw joint when chewing food, pain in and around the temples, tenderness of muscles at the side of the face, and a prolonged burning sensation in the tongue or other parts of the mouth. Prior to the start of the study, all field researchers were calibrated on performing the interview accordingly. After every question that was asked to the participant, the reviewer give a calibrated explanation to the participant regarding the meaning of the question.

The questionnaire was divided into the following sections: questions number 1 to 5 evaluated the participants’ economical and occupational aspect, questions number 6 and 7 evaluated the participant’ involvement in social activity, questions 8 to 10 evaluated the participants’ residential location, questions 11 to 13 evaluated the participants’ access to a dental health facility as well as dental health awareness, questions 14 to 16 evaluated the participants’ dental habit, questions 17 to 19 evaluated the participants’ daily habit regarding food consumption and workout habits, and questions 20-25 evaluated participants’ oral parafunctional habits, such as smoking, unilateral chewing, bruxism, excessive gum chewing, nail and lip biting as well as daytime clenching. After the completion of the twenty-five questions, the participants were asked whether they experienced or experiencing orofacial pain within the last six months. All data were then recorded and analyzed by using the Chi Square test to test the significance and were analyzed for Odds Ratio (OR) as well as Relative Risk (RR) by using *SPSS* version 23 (IBM Statistic, USA).

## 3 RESULTS

The current study recruited 1056 participants (mean age ±31 years old) who are considered to be at the productive age (18-45 years old). From the analysis of the demographical characteristics and occurrence of orofacial pain (Table **2**), it was revealed that orofacial pain mostly occur in participants who were at the 31 - 45 years old age group (52.62%), female (50.28%), elementary school graduates (55.72%), and work as laborer (69.49%). Others demographical characteristic can be viewed in Table **2**. An evaluation performed on dental related factors to the occurrence of orofacial pain can be viewed in Fig. (**2**)

Another analysis performed between these dental related factors to the occurrence of orofacial pain revealed an unusual results, where out of the 994 participants who do not have a regular dental visitation schedule, 513 (51.60%) participants have never experienced orofacial pain; and out of the 1034 participants who do not brush their teeth twice a day, about 50.29% (520) participants have never experienced orofacial pain; and that despite the fact that the nearest community health center located to their residential area has dental facilities, orofacial pain still occurred on 421 (out of 842) participants.

Significant associations between the occurrence of orofacial pain and oral parafunctional habits (bruxism (*p*<0.01), unilateral chewing (*p*<0.01), and daytime clenching (*p*<0.01)), Odds Ratio (OR) as well as Relative Risk (RR) were also calculated in the current study and can be viewed in Table **3**. One of the highest OR revealed in the study was the OR calculated to test the association between unilateral chewing to the occurrence of orofacial pain, which was 2.33, which means those who performed unilateral chewing on a regular basis, are 2.33 times more likely to have orofacial pain compared to those who chew bilaterally. It was also revealed that those who chew from one side of the mouth have a 55% increased risk (RR=1.55; 95% CI: 1.35 - 1.79) in having orofacial pain compared to those who do not chew from one side of the mouth only. A rather unusual finding regarding OR and RR were found when an association between regular dental visit twice a year and the occurrence of orofacial pain was tested. An OR of 3.34 was obtained regarding this particular association, which means those who went for a regular dental visitation twice a year are 3.34 times more likely to have orofacial pain compared to those who do not. A relative risk calculation regarding this particular association revealed that those who have a dental visitation schedule twice a year have an increased risk of 57% (RR=1.57; 95% CI: 1.34 - 1.83) in having orofacial pain compared to those who do not have a routine dental visitation schedule.

## 4 DISCUSSION

The result of the current study suggested that several oral parafunctional habits, which are bruxism, unilateral chewing, and daytime clenching were potential risk factors for the occurrence of orofacial pain. Oral parafunctional habits have been known for their short or long term effect on the oral structures. One of the oral parafunctional habits that are assumed to be closely related to the occurrence of TMD pain is bruxism, which is a type of oral parafunctional habit consists of clenching and grinding due to possible abnormalities in the masticatory system. Bruxism might be caused by psychological, morphological, as well as pathophysiologic factors [15]. A study conducted by Ahlberg *et al*, (2005) about the association between perceived orofacial pain and reported bruxism showed that orofacial pain experienced by the research participant in recent time was significantly associated to frequent bruxism [16].

Another oral parafunctional habit that was found to be significantly associated to the occurrence of orofacial pain in the current study was daytime clenching. This particular result of the current study is in line with a study that was performed to evaluate the association between several oral parafunctional habits and several diagnoses classified to TMD subgroup. In that study, daytime clenching was found to be a significant risk factor for myofascial pain, of which people who frequently did daytime clenching are 4.9 times more likely to get myofascial pain compared to those who did not do it as frequent as those who did [17]. Another study that evaluated the association between oral parafunctional habits and painful TMD showed that those who were doing two or three concomitant oral parafunctional habits were more likely to develop painful TMD [18].

Another study that showed similar result to the current study is the one that was performed by Velly *et al.*, (2003) about contributing factors of chronic masticatory myofascial pain. The result of this investigational study showed that clenching and grinding were found to be associated to masticatory myofascial pain [19]. Clenching or what is identified as an activity of bringing the teeth in contact with high level of force, causes the masticatory muscles to tighten more than normal. A repetition of this mechanism will cause an accumulation of substrates that will interfere with the intracellular pH and the conduction of action potential necessary for muscle activation [20]. This will finally lead to muscle fatigue and in the end, muscle pain.

The result of the current study also revealed a significant association between unilateral chewing to the occurrence of orofacial pain. A study by Santana-Mora *et al*, (2013) about the habitual chewing side syndrome on temporomandibular disorder revealed that habitual chewing on a certain side of the mouth is significantly associated to the painful side being complained by TMD patient [21]. Whilst a study by Reinhardt *et al*, (2006) revealed that individual with unilateral chewing habit showed more signs and symptoms of TMD [22], Another result of the current study was the significant association between heavy lifting and the occurrence of orofacial pain. Heavy lifting or weight lifting has been closely related to TMD and that the symptoms might vary from sensation of pain in the temporomandibular joint to limited jaw opening [23]. During heavy lifting or any other maximal muscular activity, voluntary clenching is somehow a very common thing to do [24]. Considering that the participants are at work in most days and repeatedly performed the heavy lifting, and as a consequence repeatedly clenched their teeth voluntarily, the occurrence of orofacial pain seems to be logically associated.

The last significant association found in the current study was the association between having a regular dental visitation schedule twice a year to the occurrence of orofacial pain. It was revealed that participants who had a regular dental visit twice a year had an increased risk of 57% of having orofacial pain compared to those who did not have regular dental check-up. This particular finding of the current study might be caused by several factors, such as participants’ overall dental condition prior to the visit. The question asked in the questionnaire did not explore when the participant started the regular dental visit, whether they have been visiting the dentist regularly for years or have just been visiting for the last year. If the later was the condition, then a poor oral health status might be the participants’ starting point. Therefore, when they started to regularly visit the dentist, they are more aware with dental and oral problems that might manifest as orofacial pain. This condition might result as if the regular dental visit was associated with the occurrence of orofacial pain. Further study exploring the association between these factors might provide clearer explanation regarding this particular result.

As for the rest of the assumed risk factors assessed in the current study (*i.e*. age, gender, educational attainment, stress), despite the fact that these factors were found to be significantly associated to the occurrence of orofacial pain in previous studies, there were no other significant association between these factors and the occurrence of orofacial pain were found in the current study. According to previous studies, [13, 25-27] there are several factors that might be potential risk factors for the occurrence of orofacial pain, such as age, educational attainment, gender, psychosocial, genetic, environment, pain sensitivity, and oral parafunctional habits. In relation to this, the current study also assessed the association between nail biting as one of the oral parafunctional habits that was found to be associated with the occurrence of orofacial pain in previous studies. Unlike the finding on previous study by Macfarlane *et al*, (2003) about the association between mechanical factors and the occurrence of orofacial pain [26], there was no association found between nail biting with the occurrence of orofacial pain. In their study, Macfarlane *et al*., revealed that nail biting is significantly associated with the occurrence of orofacial pain [26].

## CONCLUSION

To conclude, from the finding of the current study it can be summarized that the occurrence of orofacial pain in Indonesian sample might be associated to certain oral parafunctional habits, work activity, as well as regular dental visits. Further study based on the result of the current study is an avenue for future investigation. Future studies that evaluated other suspected risk factors of orofacial pain, such as genetic factor that might resulted in a more tailored treatment for an individual with orofacial pain [25] would also be of great importance. Last but not least, a study that thoroughly evaluated the involvement of psychological factor should also be considered for future investigation.

## Figures and Tables

**Fig.(2):**
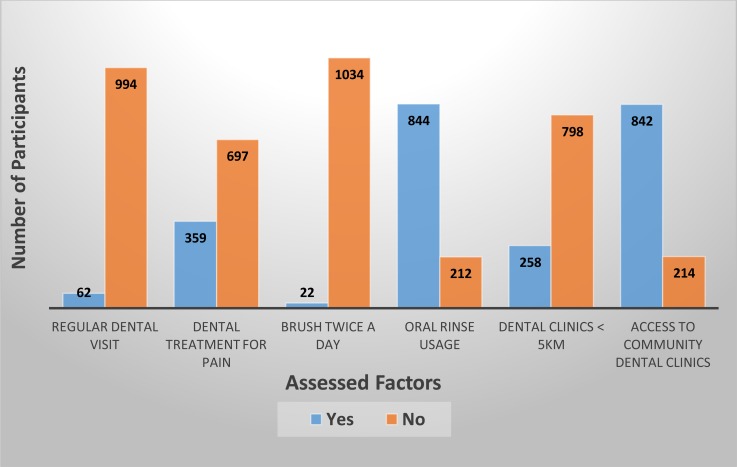
Participants distribution on dental related factors evaluation.

**Table 1:** Participant’s demographical data.

**Categories**	**Number of Participants**															
**Age**	**18 – 30 Years Old**								**31 -45 Years Old**							
	522								534							
**Gender**	**Male**				**Female**				**Male**				**Female**			
	272				250				251				283			
**Educational Attainment**	**Elementary** **School**		**Junior High** **School**				**High School**				**College** **(3 years)**				**University** **(5 years)**	
	201		304				478				32				41	
**Occupation**	1*	2*		3*		4*		5*		6*		7*		8*		9*
	192	219		81		7		359		84		42		71		1

*occupation categories: 1: private sector worker; 2: entrepreneur; 3: laborer; 4: driver; 5: housewife; 6: Government employee; 7: Student; 8: unemployed; 9: health professional.

**Table 2:** The distribution of the occurrence of orofacial pain based on demographical characteristics of the participants.

**No**	**Demographical Characteristics**	**Occurrence of Orofacial Pain**			
		**Yes**		**No**	
		Number	Percentage (within demographical characteristic)	Number	Percentage (within demographical characteristic)
**1**	**Age**				
	18 – 30 years old	247	47.32%	275	52.68%
	31 – 45 years old	281	52.62%	253	47.38%
**2**	**Gender**				
	Male	260	49.71%	263	50.29%
	Female	268	50.28%	265	49.72%
**3**	**Educational attainment**				
	Elementary school	112	55.72%	89	44.28%
	Junior High School	147	48.36%	157	51.64%
	Senior High School	243	50.84%	235	49.06%
	College (3 years)	12	37.5%	20	62.5%
	University (5 years)	14	34.15%	27	63.85%
**4**	**Occupation**				
	Private sector worker	99	51.56%	93	48.44%
	Entrepreneur	106	48.40%	113	51.60%
	Laborer	49	69.49%	32	39.51%
	Driver	2	28.57%	5	71.43%
	Housewife	185	51.53%	174	48.47%
	Government employee	34	40.48%	50	59.52%
	Student	25	59.52%	17	40.48%
	Unemployed	28	39.44%	43	60.56%
	Health professional	0	0%	1	100%

**Table 3:** Significant associations, Odds Ratios (OR), and Relative Risks (RR) for the occurrence of orofacial pain and assessed risk factors.

**No**	**Risk Factors**	***p*-value**	**OR**	**RR**
**1**	Heavy lifting at work on daily basis.	0.02	1.4	1.19 (95% CI; 1.04 - 1.35)
**2**	Regular dental visit (2x/year)	< 0.01	3.34	1.57 (95% CI; 1.34 – 1.83)
**3**	Unilateral chewing	< 0.01	2.33	1.55 (95% CI; 1.35 – 1.79)
**4**	Bruxism	< 0.01	1.6	1.26 (95% CI; 1.09 – 1.45)
**5**	Daytime clenching	< 0.01	1.9	1.33 (95% CI; 1.13 – 1.57)
